# Resection of Primary and Recurrent Parapharyngeal Space Pleomorphic Adenomas via a Combined Transcervical-Transparotid Approach: A Case Series

**DOI:** 10.7759/cureus.39700

**Published:** 2023-05-30

**Authors:** Georgios V Psychogios, Maria C Michali, Eleni V Litsou, Ioannis D Komnos, Lentiona V Basiari

**Affiliations:** 1 Department of Otorhinolaryngology, Head and Neck Surgery, University Hospital of Ioannina, Ioannina, GRC

**Keywords:** intraoperative neuromonitoring, transparotid, transvervical, pleomorhic adenoma, parapharyngeal space

## Abstract

Primary parapharyngeal space tumors are rare, and due to the complex anatomy of the parapharyngeal space, their diagnosis and treatment are challenging. Pleomorphic adenoma is the most common histologic type followed by paragangliomas and neurogenic tumors. They can present as a neck lump or an intraoral submucosal mass with the displacement of the ipsilateral tonsil or might be asymptomatic and discovered incidentally on imaging obtained for other reasons. Magnetic resonance imaging (MRI) with gadolinium is the imaging of choice. Surgery remains the treatment of choice and many approaches have been described. In this study, we present three patients with PPS pleomorphic adenoma (two primary and one recurrent), which were resected successfully with a transcervical-transparotid approach without mandibulotomy. Division of the following anatomical structures: the posterior belly of the digastric muscle, stylomandibular ligament, stylohyoid muscle and ligament, and styloglossus muscle is a very important tip for the surgeons because enables displacement of the mandible providing excellent exposure for complete tumor excision. The only postoperative complication was temporary facial nerve palsy in two patients who fully recovered within two months. The aim of this mini case series is to present our experience, together with some tips and benefits of the transcervical-transparotid approach for the resection of pleomorphic adenomas of the PPS.

## Introduction

Primary tumors of the parapharyngeal space are not common, accounting for only 0.5% of all head and neck tumors [1]. Most of them, approximately 80%, are benign neoplasms [1]. The parapharyngeal space is a complex suprahyoid anatomical space, described as an inverted pyramid with the base formed by the skull base and the apex reaching the greater cornu of the hyoid bone. It is lateral to the pharynx and contains two compartments, the prestyloid and poststyloid compartment divided from the fascia running posteriorly from the styloid process to the tensor veli palatini muscle [2]. The prestyloid compartment is located anteriorly and contains fat, the retromandibular part of the deep lobe of the parotid gland, and lymph nodes. Most neoplasms in this compartment are of salivary gland origin. The poststyloid compartment located posteriorly contains many vital structures like the internal carotid artery, internal jugular vein, cranial nerves IX, X, XI, and XII, the cervical sympathetic chain, and lymph nodes. Tumors in this region can arise from each of these structures. Controversy exists among authors regarding what is considered a true parapharyngeal space lesion. It is not accurate to consider all deep lobe parotid tumors parapharyngeal lesions. Only tumors arising from the retromandibular part of the deep lobe should be considered of parapharyngeal origin. Likewise, only carotid body paragangliomas located above the posterior belly of the digastric muscle should be considered parapharyngeal space masses [3].

The differential diagnosis of benign PPS tumors includes pleomorphic adenomas of the salivary glands, which are the most common, followed by paragangliomas and neurogenic tumors [1]. PPS tumors usually present very few symptoms and are diagnosed only when they become large enough to be detected. More often they present as an intraoral smooth submucosal mass displacing the tonsil and the soft palate but sometimes, they can also appear as a neck mass [4]. Imaging studies play a key role in diagnosis and are used to predict the origin, location, and size of parapharyngeal tumors and for preoperative planning. Gadolinium contrast-enhanced MRI and computed tomography (CT) with contrast are the most common imaging tools used for parapharyngeal space tumor evaluation. In cases when imaging shows a widening of the carotid bifurcation known as the Lyre sign or when a vascularized mass is denoted by an early contrast enhancement CT, angiography is recommended [5].

Surgical excision remains the best treatment for PPS tumors. Different surgical approaches have been described, including transcervical, transparotid, transoral, infratemporal, and transmandibular [6]. Alone or in combination, these approaches have been used to resect PPS tumors depending on the size, location, relationship with the surrounding vessels and nerves, and surgeon’s preference.

In this article, we present three cases of parapharyngeal space pleomorphic adenomas resected with a combined transcervical-transparotid approach without mandibulotomy, together with some tips for better visualization of the tumor with this approach.

## Case presentation

Case 1

**Figure 1:**
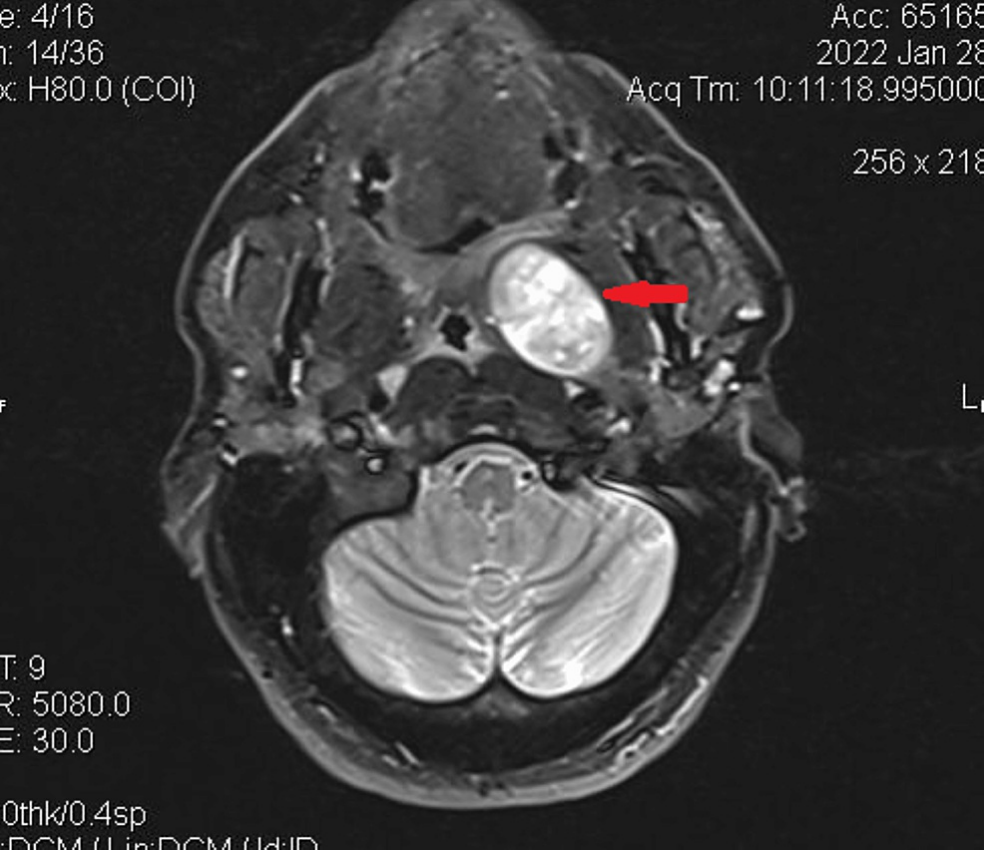
MRI imaging (T1 transverse) showing the parapharyngeal space mass with the displacement of the left tonsil and lateral pharyngeal wall (red arrow)

A 40-year-old male patient presented to our department two years ago with a left parapharyngeal space tumor that was found incidentally on a pituitary MRI, which he underwent because of pathological prolactin levels. He also complained of snoring and sleep apnea. MRI of the neck revealed a tumor of the left parapharyngeal space with benign characteristics and dimensions 3.9 Χ 3.9 Χ 2.7 cm, which caused narrowing of the oropharynx (Figure 1)

During physical examination, there was no palpable neck mass but there was an evident bulging of the left lateral pharyngeal wall causing displacement of the left tonsil and left soft palate. The tumor was resected through a transcervical-transparotid approach and a biopsy showed a pleomorphic adenoma. The patient had a temporary facial nerve palsy (House-Brackmann II) postoperatively, which fully recovered (HB I) after one month, and he also mentioned improvement in snoring and sleep apnea in the next postoperative months. The patient was on regular follow-up postoperatively and the latest follow-up was six months ago (almost 1.5 years after the operation) with an MRI that showed no signs of recurrence.

Case 2

**Figure 2:**
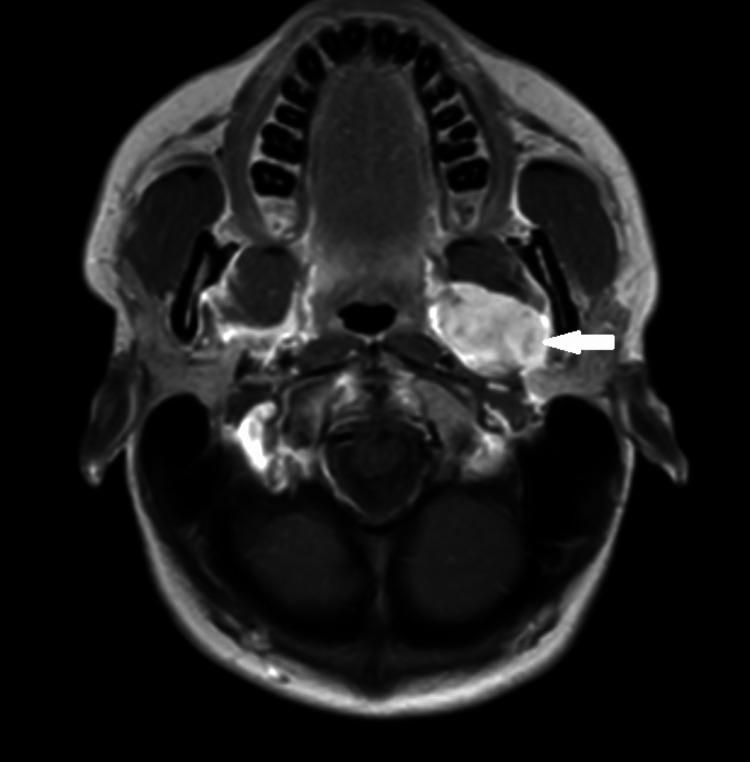
MRI (T1 transverse) showing the tumor in the parapharyngeal space (white arrow)

A 30-year-old woman presented to our department a year ago with a left parapharyngeal space tumor, of dimensions 3.1 X 2.6 X 3.4 cm, which was found incidentally on a head and neck MRI that she underwent due to episodes of syncope. The possible diagnosis from the MRI was a neurogenic tumor (Figure 2).

From the clinical examination, there was no evident neck mass nor an intraoral mass. The tumor was resected through a transervical-transparotid approach, and the pathology report with immunohistochemistry showed a pleomorphic adenoma. The tumor was resected with its capsule intact, and no frozen sections were performed intraoperatively. Postoperatively, the patient presented temporary facial nerve palsy (House-Brackmann IV), which fully recovered within two months. She was discharged from the hospital in three days.

Case 3

**Figure 3:**
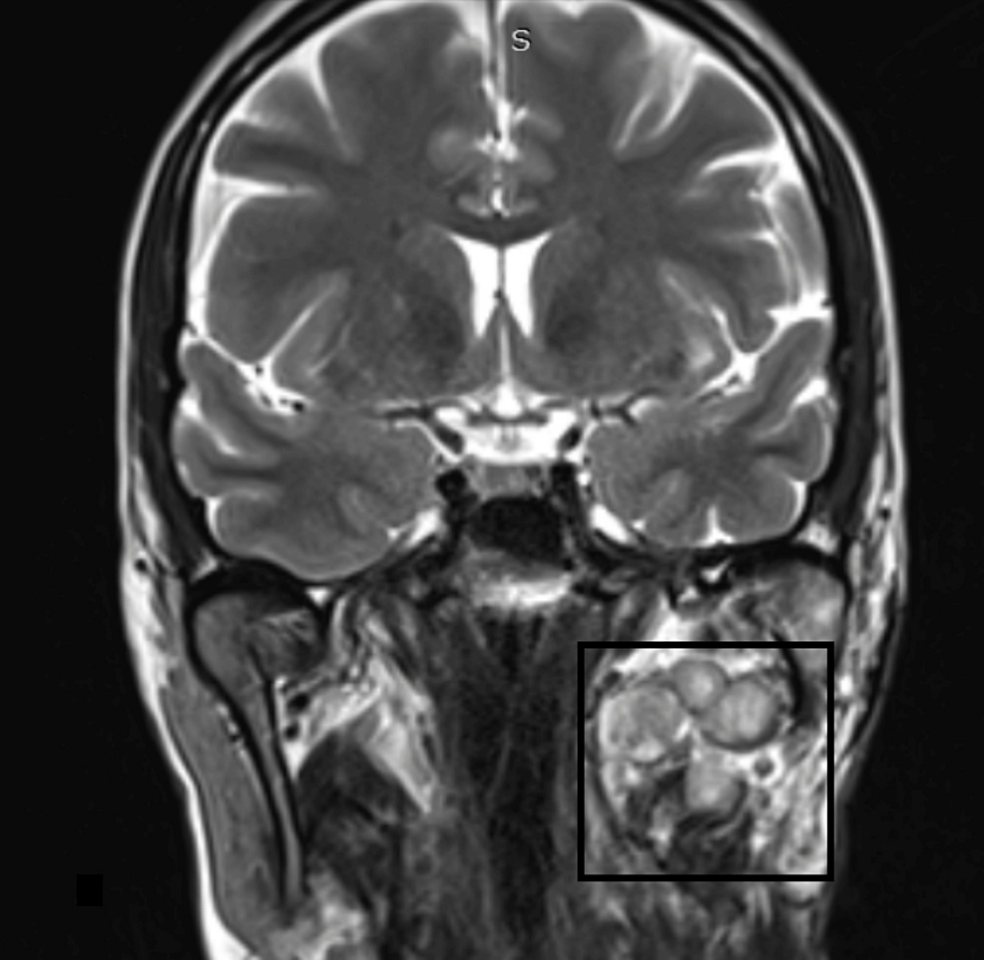
MRI coronal view revealing the multinodular recurrence of the pleomorphic adenoma (black square)

A 37-year-old woman with a repeatedly recurrent pleomorphic adenoma of the parapharyngeal space presented to our department 8 months ago. She had undergone three previous surgeries during the last 10 years in other hospitals. According to her medical record, she suffered from thrombophilia. Preoperatively, a complete investigation of the coagulation profile was performed with normal INR, PT, and aPTT values, and in addition, a consult with the hematologist was done. MRI showed a multinodular recurrent pleomorphic adenoma (Figure 3).

**Figure 4:**
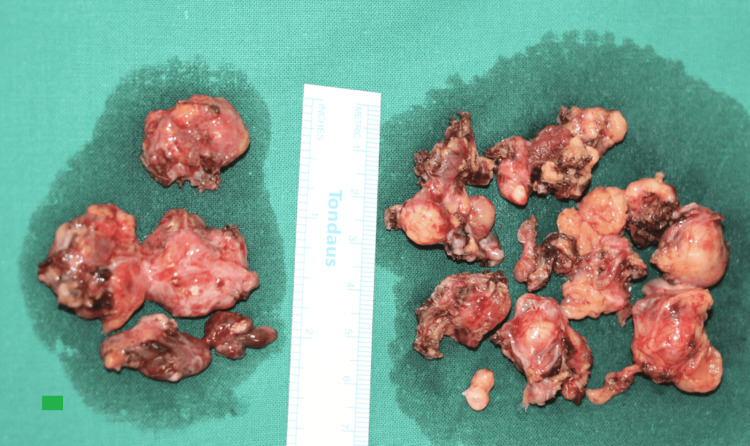
The resected nodules of the recurrent pleomorphic adenoma

The transcervical-transparotid approach was also used in this case, and we managed to resect approximately more than 10 separate nodules of pleomorphic adenoma (Figure 4).

**Figure 5:**
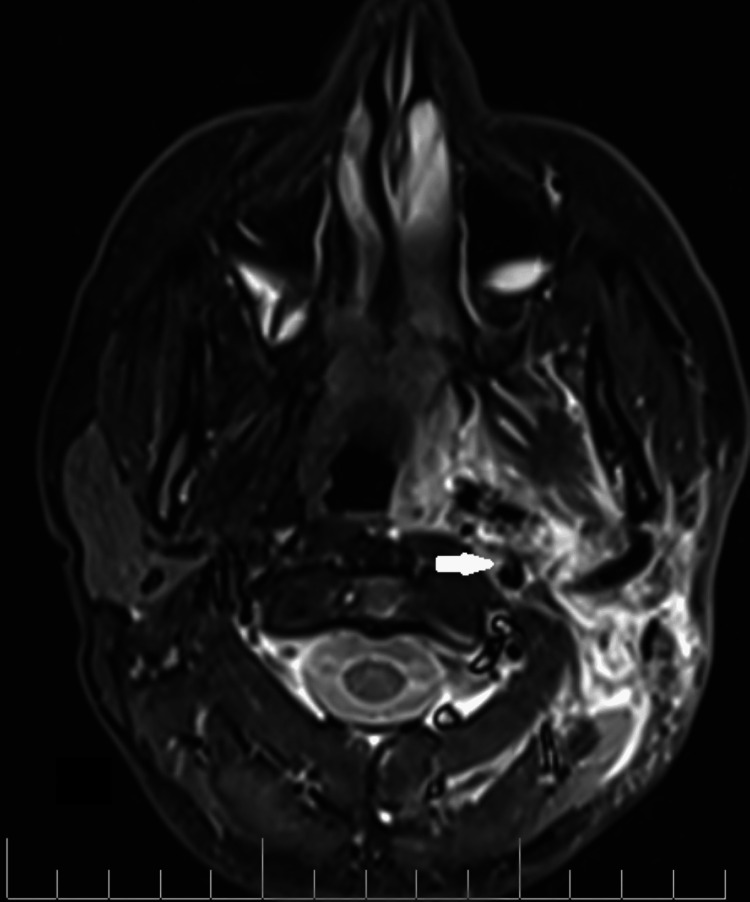
Postoperative MRI showing the parapharyngeal space (white arrow) with postoperative changes and no signs of tumor

The patient was hospitalized for three days, and there were no postoperative complications. A magnetic resonance 10 days after the surgery showed no signs of residual tumor in the PPS (Figure 5).

Although in this case, the probability of recurrence remains high, we managed to successfully resect the tumor and, most importantly, preserve the facial nerve, which is at great risk of injury during reopening for recurrence. The most recent follow-up was six months postoperatively, and the patient is scheduled for an MRI in the next months.

Surgical technique

**Figure 6:**
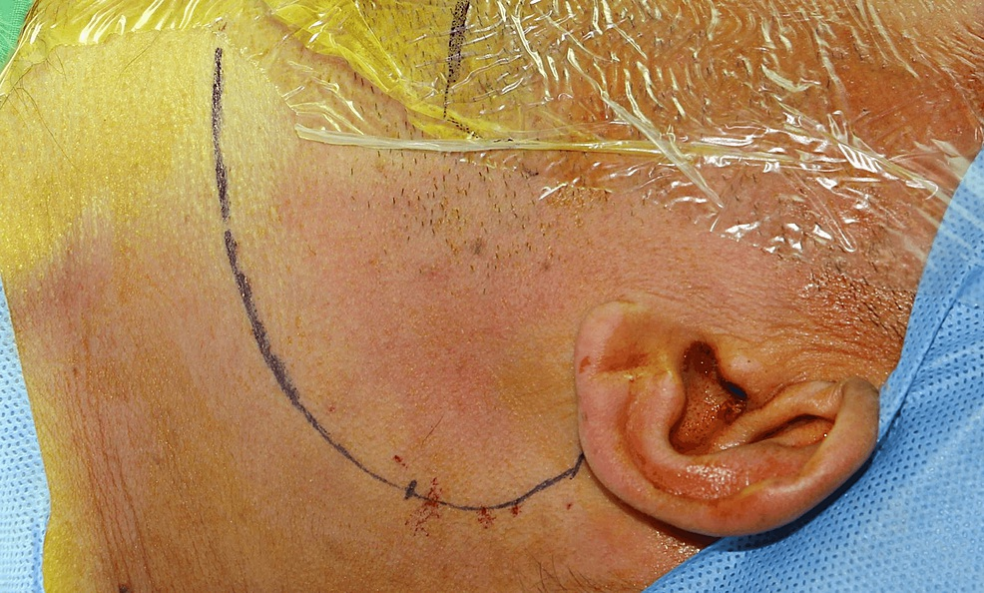
Skin incision used for transcervical-transparotid approach

In the three cases during the operation of intraoperative neuromonitoring for the facial nerve (Medtronic NIM III; Medtronic plc, Minneapolis, Minnesota) was used. A skin incision was performed as shown in Figure 6, and skin flaps were raised in a subplatysmal plane with caution not to damage the marginal mandibular branch of the facial nerve, which was recognized using the intraoperative neuromonitoring.

**Figure 7:**
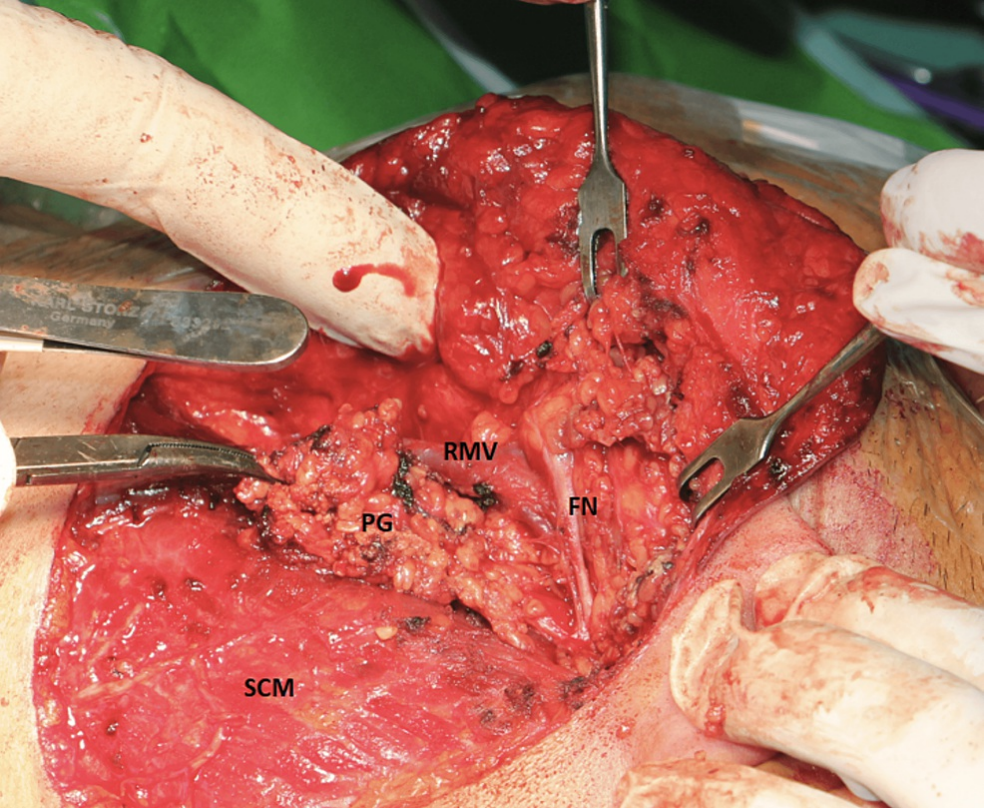
Facial nerve (FN) after exiting the stylomastoid foramen and entering the parotid gland In the figure, the caudal part of the superficial part of the parotid gland (PG) has been mobilized in order to be excised. (SCM: sternocleidomastoid muscle, RVM: retromandibular vein)

Next, the main trunk of the facial nerve was recognized while exiting from the stylomastoid foramen (Figure 7).

The superficial lobe was mobilized, and its caudal portion (MSGS level II) was resected in order to expose the deep lobe of the gland. With caution, the caudal part of the deep lobe (MSGS level III) was also resected. The posterior belly of the digastric muscle, the stylomandibular ligament, the stylohyoid muscle and ligament, and the styloglossus muscle were recognized and divided. This step of the procedure is very important because it enables the displacement of the mandible upward with a retractor, providing a much better surgical field and access to the parapharyngeal space. The submandibular gland was also mobilized and retracted upward and part of the fat of the submandibular compartment was resected to improve access. It is not necessary to resect the submandibular gland. The facial vein was ligated. The common carotid artery with its bifurcation was recognized, and caution was shown not to damage the internal carotid artery by pressing it on the styloid process during the resection of the tumor near the skull base. The internal jugular vein was preserved. The hypoglossal, accessory, and glossopharyngeal nerves were recognized and protected. For confirmation, we used the neuromonitoring system and delivered electrical pulses to the nerves, especially the hypoglossal and accessory nerves, and we noticed the contraction of the muscles of the tongue and the shoulder.

**Figure 8:**
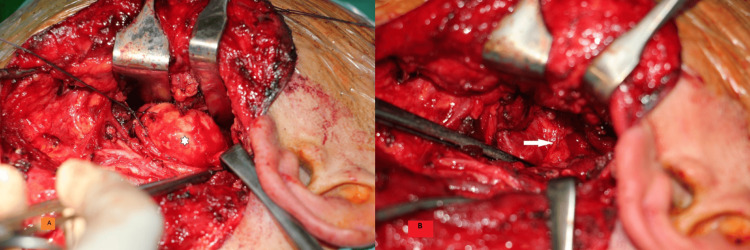
A) After the division of the posterior belly of the digastric muscle, stylomandibular ligament, stylohyoid muscle and ligament, and styloglossus muscle, the mandible is retracted as shown, and after blunt dissection, the tumor (white star) with its capsule intact is resected. B) View of the entrance to the parapharyngeal space after tumor excision (white arrow)

The dissection reached the skull base superiorly and the superior constrictor pharyngeal muscle medially. The position of the internal carotid artery and the glossopharyngeal nerve were continuously monitored during these last steps of the surgery. Then, the tumor was resected with blunt dissection with the capsule intact (Figures 8A, 8B).

Copious irrigation of the wound is important if there is any tumor spillage. Finally, drain insertion and layered closure were performed. In the third case, because it was a reoperation, it was more difficult to recognize the facial nerve, which was not in its usual place, and there were multiple nodules that should be resected, which prolonged the time of the operation and increased the risk for complication.

## Discussion

The parapharyngeal space is one of the most anatomically complex spaces of the neck. Tumors of varying histopathologies develop in this region, with most of them of benign nature. Due to the complex anatomical relationships and histological diversity, the diagnosis and treatment of PPS tumors present a challenge for physicians. It is helpful in determining the possible diagnosis to distinguish whether a lesion arises in the prestyloid or the poststyloid compartment. As a general rule, prestyloid lesions usually displace the internal carotid artery (ICA) posteriorly while poststyloid lesions displace the ICA anteromedially [7]. Most prestyloid tumors arise from the salivary glands with pleomorphic adenomas being responsible for 65% of all salivary gland neoplasms [1]. In our study, the diagnosis for the three patients was pleomorphic adenoma, primary in the two first cases and recurrent in the third patient. Other benign tumors of the parapharyngeal space include paragangliomas (carotid body tumors or glomus vagale), schwannomas, and neurofibromas. Paragangliomas are usually sporadic but can also be associated with syndromes. Schwannomas are usually vagal or sympathetic chain in origin and can cause dysfunction of cranial nerves IX, X, and XII due to mass effect. Surgical excision of enlarging neurogenic lesions is recommended, and it is usually associated with dysfunction of the nerve of origin after resection [8].

Regarding clinical presentation, benign PPS tumors usually present as a neck mass or an intraoral swelling causing asymmetry of the ipsilateral tonsil, soft palate, and lateral pharyngeal wall. They can cause symptoms like snoring, glomus sensation, dysphagia, and dysphonia related to mass effect and impaired vagal nerve function. It is not uncommon though for patients to present with a parapharyngeal tumor that was incidentally discovered on imaging obtained for other reasons. Symptoms like otalgia, facial pain, and trismus should raise suspicion of malignancy [9]. In our study, though the first patient complained of snoring and sleep apnea, the tumor was discovered incidentally on a pituitary MRI. In the second patient, it was also an incidental finding, and in the third case, it was discovered during a follow-up MRI.

Imaging studies are crucial for the diagnostic workup of these tumors and are used to predict the origin, side, and size of parapharyngeal tumors. Pre-operative assessment typically includes an MRI and/or CT scan. Angiography is required in cases of vascular lesions. MRI with gadolinium is the examination of choice because it can be typical for many lesions of the parapharyngeal space. Usually on MRI examination, salivary gland neoplasms are typically T2 hyperintense, and it is possible to recognize a fat plane between the parotid and the mass while schwannomas are typically T1 isointense or hypointense, with enhancement during the administration of gadolinium [7,10]. In our study, MRI was the imaging of choice. Regarding fine needle aspiration (FNA) biopsy, although intraoral or transcervical FNA is accurate in 90-95% of cases and can be performed under CT or ultrasound guidance, it has been reported that in 25-60% of cases, it is not diagnostic due to lack of cellular material, bleeding or other technical issues [10-12].

Most patients with parapharyngeal space masses are treated with surgery and the aim of surgery is to entirely remove the lesion with minimal postoperative complications. The selected surgical approach must be wide enough to guarantee the complete removal of the tumor and to prevent injury to the vessels and nerves of the upper neck [10]. Many surgical approaches have been described in the literature [13]. Factors like tumor location inside the parapharyngeal space, tumor dimensions and relations with nearby vital structures, and the possibility of malignancy dictate the choice of the surgical approach [14,15].

The transcervical approach is most appropriate for the resection of small tumors of the inferior part of the parapharyngeal space [15].

The transcervical-transparotid approach is recommended for tumors adjacent to the facial nerve such as deep-lobe parotid tumors and minor salivary gland tumors. Through this approach, it is not only possible to identify and preserve the facial nerve but also the external and internal carotid arteries, the internal jugular vein, the cranial nerves IX, X, XI, XII, and the sympathetic nervous system chain. Although a superficial parotidectomy might be necessary to be performed to identify and carefully preserve the facial nerve, an effort is made to preserve the superficial lobe of the parotid gland in order to reduce the risk of developing Frey’s syndrome and for better cosmesis [9, 15,16]. This approach may also be appropriate for poststyloid lesions, like neurogenic tumors located in the mid to upper portion of the parapharyngeal space [15]. For large parapharyngeal space tumors reaching the skull base, the mandible represents a significant anatomical obstacle for adequate exposure. In order to displace the mandible upward with a retractor to achieve better exposure a valuable tip is to divide the posterior belly of the digastric muscle, the stylomandibular ligament, the stylohyoid muscle and ligament, and the styloglossus muscle. A mandibulotomy might also be performed, but it is better to be avoided for benign tumors because it has been associated with higher morbidity, and it may require a covering tracheostomy, a nasogastric feeding tube, longer hospital stay, and a delay in oral nutrition [15]. It has also been related to an increased risk of mandibular malunion and temporomandibular joint dysfunction postoperatively [15,17]. In our three patients, we performed a transervical-transparotid approach, and we managed to safely resect the tumors achieving good exposure. In addition, the submandibular gland was retracted upward and not resected. We did not resect the entire superficial lobe of the parotid gland but only part of it and the use of intraoperative monitoring helped us minimize the risk of facial nerve damage. Postoperatively, we had only temporary paralysis of the facial nerve in the first two patients, which recovered gradually. Pleomorphic adenomas of the parapharyngeal space are characterized by a thicker and stronger capsule which allows blunt dissection without great risk of intraoperative injury to the capsule or to the nearby important anatomical structures [18].

The transoral approach has also been described but most authors do not recommend routine use of this technique because the limited exposure has been related to a higher risk of tumor rupture, incomplete removal, facial nerve injury, fistula, and uncontrollable hemorrhage [15]. However, in selected cases, and in combination with a transcervical approach, benign tumors of the parapharyngeal space have been resected successfully without any major complications [19].

Transoral robotic surgery (TORS) has been used in recent years to resect small benign lesions of the parapharyngeal space with promising results. Supporters of the approach claim that TORS provides a better three-dimensional view of the surgical area and safer tumor dissection but due to the limited availability of the expensive equipment and the potential for complication, there are very few centers worldwide that perform this approach [15,20].

In conclusion, an optimal surgical approach should be the safest and most effective method for providing complete tumor removal and preventing injury to the vessels and nerves of the parapharyngeal space. Intraoperative visibility of all vital anatomical structures is essential in performing a safe and radical dissection of the tumor. On the other hand, the surgical approach should also advocate providing minimal functional and cosmetic side effects.

## Conclusions

Parapharyngeal space tumors are rare, and their diagnosis and treatment are challenging due to the complex anatomy of the region. They may present with few clinical symptoms or can be entirely asymptomatic until reaching large dimensions. Asymptomatic tumors are usually diagnosed incidentally on radiological examinations of the head and neck for other reasons. The majority are benign tumors and arise from the salivary glands. Pleomorphic adenoma is the most common histologic type. Surgery is considered the treatment of choice after a complete investigation. Surgical strategy is determined by location, size, and pathology. In this mini case series, we demonstrate that the transervical-transparotid approach with a division of the following anatomical structures: stylomandibular ligament, posterior belly of the digastric muscle, stylohyoid muscle and ligament, and styloglossus muscle provides excellent exposure and enables complete excision of primary and recurrent parapharyngeal space pleomorphic adenomas with minimal morbidity. The use of intraoperative nerve monitoring is very important, and we recommend it through this approach for preventing facial nerve damage.
